# Early Multicenter Outcomes of Dedicated Transcatheter Aortic Valve Replacement for Left Ventricular Assist Device‐Associated Pure Aortic Regurgitation

**DOI:** 10.1111/aor.70226

**Published:** 2026-08-25

**Authors:** Hristian Hinkov, Sara Yousefzada, Isabell A. Just, Christoph Klein, Evgenij Potapov, Santiago Garcia, Dhairya Patel, Lauren S. Ranard, Arseniy Goncharov, Dean J. Kereiakes, Raj R. Makkar, Torsten P. Vahl, Tanja K. Rudolph, Axel Unbehaun

**Affiliations:** ^1^ Department of Cardiothoracic and Vascular Surgery Deutsches Herzzentrum der Charité (DHZC) Berlin Germany; ^2^ Charité‐Universitätsmedizin Berlin Corporate Member of Freie Universität Berlin and Humboldt‐Universität Zu Berlin Berlin Germany; ^3^ Structural Heart Interventions Program (SHIP) Deutsches Herzzentrum der Charité (DHZC) Berlin Germany; ^4^ DZHK (German Center for Cardiovascular Research), Partner Site Berlin Berlin Germany; ^5^ Department of General and Interventional Cardiology and Angiology, Heart and Diabetes Center NRW Ruhr University Bad Oeynhausen Germany; ^6^ Department of Cardiology, Angiology and Intensive Care Medicine Deutsches Herzzentrum der Charité (DHZC) Berlin Germany; ^7^ Division of Cardiology The Christ Hospital Heart and Vascular Institute and the Carl and Edyth Lindner Center for Research and Education Cincinnati Ohio USA; ^8^ Department of Cardiology, Smidt Heart Institute Cedars‐Sinai Medical Center Los Angeles California USA; ^9^ Division of Cardiology Columbia University Irving Medical Center New York New York USA

**Keywords:** aortic regurgitation, JenaValve trilogy, left ventricular assist device, transcatheter aortic valve replacement

## Abstract

**Background:**

Aortic regurgitation (AR) is a frequent and clinically relevant complication in patients with left ventricular assist devices (LVAD), impairing circulatory support and worsening outcomes. Surgical aortic valve replacement often carries prohibitive risk in this population, while conventional transcatheter aortic valve replacement (TAVR) is limited by the lack of annular calcification. The JenaValve Trilogy is the first transfemoral TAVR system with regulatory approval for AR. Evidence on its use in LVAD patients remains scarce.

**Methods:**

We conducted a retrospective multicenter registry of LVAD recipients undergoing JenaValve Trilogy implantation for pure AR between 2020 and 2026 at five high‐volume centers in Germany and the United States. Patient characteristics, procedural details, and outcomes were analyzed. The primary endpoint was device success according to Valve Academic Research Consortium‐3 (VARC‐3) criteria at 30 days. Secondary endpoints included peri‐procedural complications, mortality, and device‐related reinterventions at follow‐up.

**Results:**

Thirty patients (median age 66 years, 36.7% female, median LVAD support time: four years) were included. One patient did not meet criteria for technical success, and one experienced a fatal hemorrhagic stroke on postoperative day 2, rendering device success 93.3% (28/30). 30‐day mortality was 3.3% (1/30). At a median follow‐up of 273 days (IQR 30–491), survival was 93.3% (28/30).

**Conclusion:**

This first multicenter experience demonstrates that JenaValve Trilogy provides a feasible and safe treatment option for clinically significant AR in LVAD patients at prohibitive surgical risk, with high device success and favorable short‐term outcomes.

## Graphical abstract

LVAD‐associated aortic regurgitation impairs circulatory support, with limited surgical and off‐label options. In a multicenter registry (*n* = 30), the AR‐dedicated JenaValve Trilogy TAVR eliminated AR, achieving 96.7% (29/30) technical success and 93.3% (28/30) device success (VARC‐3), with 3.3% (1/30) 30‐day mortality.

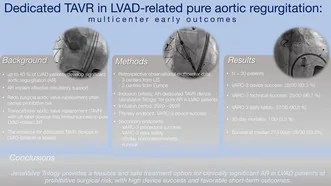

**Abbreviations**
AR: aortic regurgitation
CT: computed tomography
DLZ: device landing zone
HM3: HeartMate 3
LVAD: left ventricular assist device
NYHA: New York Heart Association
SAVR: surgical aortic valve replacement
TAVR: transcatheter aortic valve replacement
VARC‐3: Valve Academic Research Consortium 3

## 1 Introduction

Left ventricular assist devices (LVADs) have significantly improved outcomes in patients with end‐stage heart failure [1]. Their effectiveness relies on preserved aortic valve competence, however, significant aortic regurgitation (AR) is a frequent and clinically relevant complication, developing de novo or progressing from pre‐existing lesions in up to 40% of patients during long‐term support, although rates appear lower with contemporary centrifugal devices. The incidence of AR surpasses that of the commonly known LVAD‐associated complications—gastrointestinal bleeding, ischemic and hemorrhagic strokes, and driveline infections—and negatively affects outcomes [2, 3, 4, 5, 6]. AR creates a closed‐loop circuit in which blood regurgitates into the left ventricle and is repeatedly pumped through the LVAD, reducing effective systemic output, impairing end‐organ perfusion and contributing to right‐heart failure, and worsening patients' functional class [7].

Surgical aortic valve replacement (SAVR) carries a Class I recommendation for pure AR in both the 2020 AHA/ACC and 2025 ESC/EACTS guidelines, with the latter for the first time introducing transcatheter aortic valve replacement (TAVR) as a Class IIb option in anatomically suitable patients who are not surgical candidates [8, 9]. However, SAVR is frequently a suboptimal treatment option in this high‐risk population due to the cumulative risks of redo surgery, general anesthesia, cardioplegic heart arrest, bleeding, and perioperative right ventricular impairment, ultimately rendering LVAD patients unsuitable for redo surgery [10]. Heart transplantation is another treatment option, but a high number of LVAD patients are supported as destination therapy. Off‐label TAVR strategies using commercially available valves have shown feasibility but remain limited by the absence of annular calcification, increasing the risk of malposition, paravalvular leak, and valve embolization [11, 12, 13]. Although device landing zone pre‐stenting has been proposed to improve anchoring in selected anatomies, the technique is technically demanding and has not been widely adopted [11, 14]. The JenaValve Trilogy is a third‐generation, AR‐dedicated self‐expanding, transfemoral TAVR device with Conformiteé Européene (CE) regulatory approval since May 2021 and U.S. Food and Drug Administration (FDA) premarket approval since March 2026. Its leaflet‐clipping mechanism, based on three locators grasping the native aortic valve cusps from inside the sinuses of Valsalva to the main TAVR frame, facilitates fixation independent of calcification, addressing the limitations of conventional TAVR in this setting [15].

This multicenter registry study provides the largest available cumulative evidence to date on procedural feasibility and short‐term outcomes for JenaValve Trilogy implantation in LVAD patients with clinically relevant pure AR.

## 2 Methods

### 2.1 Patients

This multicenter retrospective registry included consecutive patients with durable LVAD who underwent TAVR using the JenaValve Trilogy system for significant pure native AR, defined as isolated AR without concomitant aortic stenosis and without any aortic valve calcification. Patients were treated between October 2020 and January 2026 across five tertiary centers, two in Germany (Deutsches Herzzentrum der Charité, Berlin and Heart and Diabetes Center North Rhine‐Westphalia, Bad Oeynhausen) and three in the United States (Columbia University Irving Medical Center, New York; Cedars‐Sinai Medical Center, Los Angeles and The Christ Hospital, Cincinnati). Cases included in the industry‐sponsored JenaValve JENA‐VAD/JenaValve ALIGN‐AR LVAD United States registry were not included in this work. In all cases, the local heart team deemed patients unsuitable for SAVR due to prohibitive surgical risk. The study protocol was approved by the responsible institutional ethics committee (EA4/246/25), and the requirement for individual informed consent was waived.

### 2.2 Data Collection

Collected data on patient demographics and baseline characteristics included age, sex, type of LVAD, date of LVAD implantation, the time interval between LVAD implantation and TAVR, comorbidities, surgical risk scores (EuroSCORE II) for SAVR as a redo procedure, New York Heart Association (NYHA) functional class, and contrast computed tomography (CT) derived sizing and TAVR implant planning analysis data. Procedural variables included the date of JenaValve Trilogy implantation, TAVR urgency and access, anesthesia regimen, procedural duration, contrast amount, fluoroscopy time, the need for a second transcatheter heart valve as a bail‐out valve‐in‐valve implantation, conversion to SAVR, severity of residual AR, and new‐onset cardiac rhythm and conduction disturbances. Postprocedural data included Valve Academic Research Consortium‐3 (VARC‐3) defined endpoints [16] for technical and device success, early safety, postoperative complications, device‐related rehospitalization or reintervention, length of hospital stay, and 30‐day mortality. Survival follow‐up data were collected where available.

### 2.3 Statistical Analysis

Baseline, procedural, and outcome data were summarized using descriptive statistics with means and 25%–75% interquartile ranges. Survival probabilities were assessed with Kaplan–Meier analysis, median follow‐up with inverse Kaplan–Meier, and corresponding curves were generated to visualize survival over time. All statistical analyses and their respective graphical presentations were performed using GraphPad Prism (version 10.6.0, GraphPad Software, San Diego, California, United States). A subgroup descriptive analysis of patients supported with the HeartMate 3 (HM3) LVAD was performed to assess the applicability of the findings to patients receiving currently implanted durable LVAD systems. No formal statistical comparison between HeartMate 3 and legacy LVAD platforms was performed. The HM3 subgroup results are presented in the Supplementary Table 3.1, 3.2, 3,3 and Supplementary Figure 3 (S4).

### 2.4 TAVR Sizing and Implantation Technique

TAVR procedures were performed using the JenaValve Trilogy transcatheter heart valve system in accordance with the manufacturer's instructions for use. Valve sizing was determined based on pre‐procedural end‐systolic 4D CT of the aortic annulus, with reference to the JenaValve Trilogy sizing chart for non‐LVAD‐associated AR. Annular diameter and perimeter were evaluated to select the appropriate valve size (23 mm/S, 25 mm/M, or 27 mm/L), ensuring safe anchoring to the native cusps. Implantation was performed via the transfemoral route or alternative peripheral vascular access (off‐label). The anesthesia strategy (general anesthesia or conscious sedation) was individualized according to clinical factors and the implanter's preference. During the procedure, and especially at the moment of valve deployment, LVAD flow can be kept constant since the JenaValve Trilogy design limits the probability of ventricular migration. Rapid pacing is also not generally required, further reducing the risk of right ventricular compromise. Figure 1 and Supplementary Video 1 (S1) depict the implantation sequence of JenaValve Trilogy TAVR in an LVAD patient.

**FIGURE 1:**
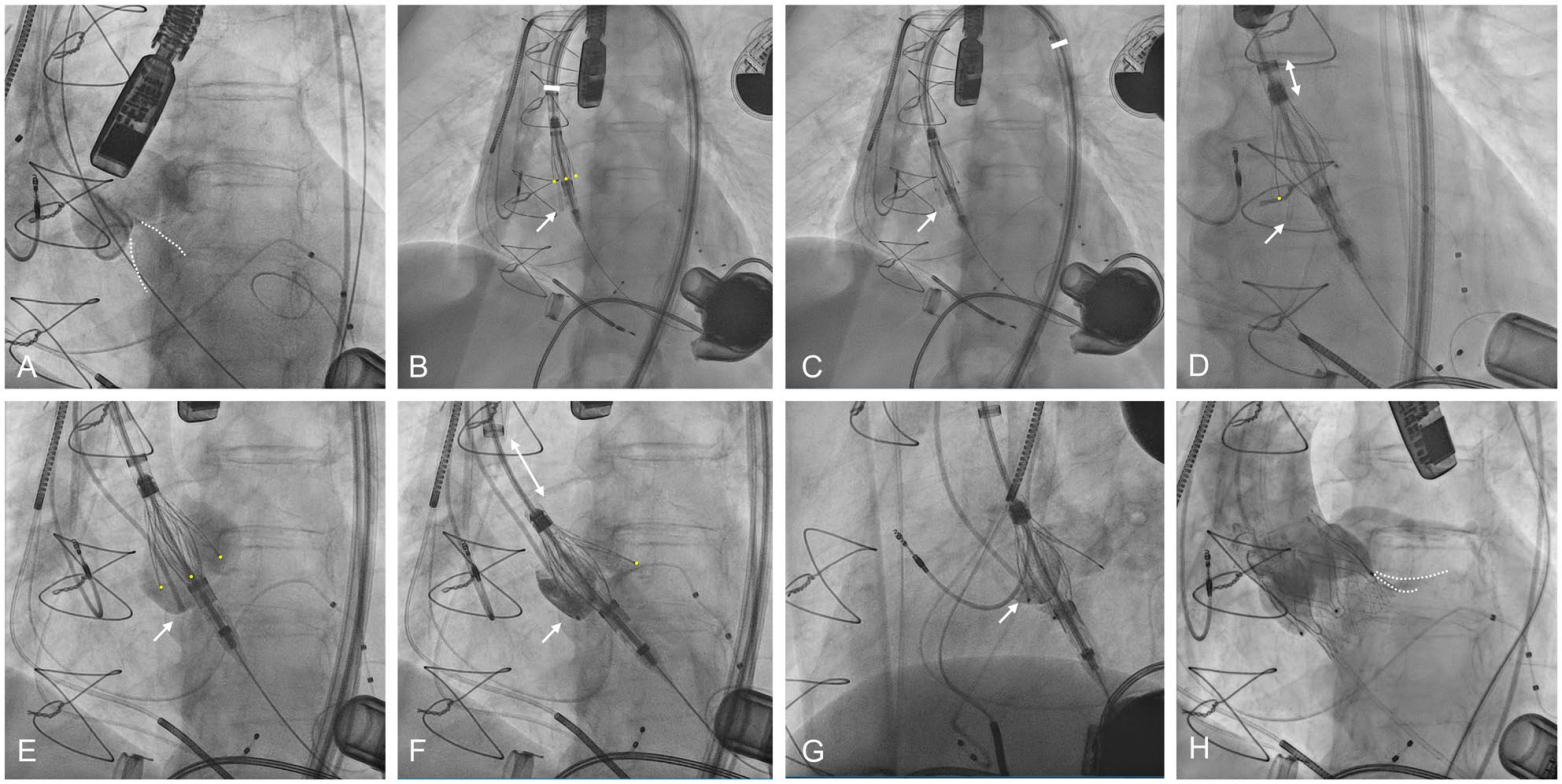
Implantation sequence of JenaValve Trilogy in a patient on a durable LVAD. (A) Baseline aortic root angiography with significant AR jet (interrupted white lines). (B) JenaValve Trilogy delivery sheath advanced to the level of the sinotubular junction (white bar). Delivery catheter and valve with 3 locators (yellow dots) positioned in the aortic root. Fluoroscopic confirmation of locator position using a multipurpose catheter (white arrow). (C) Retraction of the delivery sheath in the proximal descending aorta (white bar). (D) Advancement of the valve over the delivery catheter (double‐sided white arrow) with flaring of the locator in the noncoronary sinus (yellow dot). Multipurpose catheter in the noncoronary sinus (white arrow). (E) The locator alignment (yellow dots) to the bottom of the respective sinuses is checked angiographically. Valve positioning is depicted as too high. (F) Further advancement of the valve over the delivery catheter (double‐sided white arrow). On angiography, correct placement of the noncoronary sinus locator plus flaring of the left coronary cusp locator (yellow dot) can be appreciated, suggesting correct implantation depth. (G) Final verification of all 3 locators prior to deployment. (H) Post‐implantation root angiography confirming elimination of AR. A trace of paravalvular leak is present on the side of the left coronary cusp (interrupted white lines).

## 3 Results

### 3.1 Cohort Characteristics

The baseline characteristics of the study group are summarized in Table 1. All patients presented with clinically relevant moderate‐to‐severe or severe, symptomatic AR of a native aortic valve. Supplementary Table 1 (S2) provides patient‐level baseline data, including a detailed list of comorbidities.

**TABLE 1:** Cohort characteristics.

Cohort characteristics		*n* = 30
Age, *years*	median (IQR)	66 (58.1 | 71.0)
Gender, *female*	n (%)	11 (36.7)
BMI, *kg/m* ^*2*^	median (IQR)	26.5 (25.5 | 28.5)
Time on LVAD, *years*	median (IQR)	4 (2.0 | 4.5)
Type of LVAD	n (%)	
*HeartMate II*		3 (10)
*HeartMate 3*		20 (66.7)
*HeartWare HVAD*		7 (23.3)
EuroSCORE II, *%*	median (IQR)	9.63 (8.34 | 16.76)
NYHA functional class	*n* (%)	
*I*		0 (0.0)
*II*		3 (10.0)
*III*		16 (53.3)
*IV*		11 (36.7)
Permanent pacemaker	*n* (%)	21 (70.0)

*Note:* The Summary of the baseline cohort characteristics.

Abbreviations: IQR—interquartile range upper/lower quartile; BMI—body mass index; LVAD—left‐ventricular assist device; NYHA—New York Heart Association.

### 3.2 Procedural Characteristics and Outcome

Procedural characteristics and outcomes are presented in Table 2. Eleven out of 30 (36.7%) TAVR procedures were performed urgently and 19/30 (63.3%) electively. Urgent procedures were defined as those performed in the setting of ongoing cardiogenic shock and/or inotropic support. Transfemoral access was used in 29 (96.7%) patients. In one (3.2%) patient, transcarotid access was chosen. There were no conversions to open heart surgery in the cohort. The VARC‐3‐defined composite endpoint for technical success was 96.7% (29/30). One patient (3.3%) required immediate implantation of a second TAVR device as a bailout valve‐in‐valve procedure due to severe paravalvular leak.

**TABLE 2:** Procedure characteristics.

Procedure characteristics		*n* = 30
Device size, *mm*	*n* (%)	
23/S		3 (10.0)
25/M		11 (36.7)
27/L		16 (53.3)
Device access	*n* (%)	
*transfemoral*		29 (96.7)
*other*		1 (3.3)
Annulus perimeter, *mm*	median (IQR)	78.6 (74.5 | 82.7)
Annulus average diameter, *mm*	median (IQR)	25.7 (22.6 | 26.3)
Anesthesia regimen	*n* (%)	
*general anesthesia/intubation*		22 (73.3)
*conscious sedation*		8 (26.7)
Procedure duration, *min*	median (IQR)	67.0 (57.0 | 82.5)
Fluoroscopy duration, *min*	median (IQR)	17.1 (14.8 | 24.1)
Contrast volume, *mL*	median (IQR)	102 (76.5 | 124.0)
Technical success	*n* (%)	29 (96.7)

*Note:* The Summary of the JenaValve Trilogy TAVI implantation procedural characteristics.

Abbreviation: IQR—interquartile range upper/lower quartiles.

### 3.3 In‐Hospital Outcomes and 30‐Day Mortality

Table 3 summarizes the in‐hospital outcomes at discharge, including the VARC‐3 30‐day composite endpoints of device success and early safety. Device success and early safety were not achieved in one patient who failed to meet criteria for technical success, and in another who experienced a fatal hemorrhagic stroke on postoperative day two. A third patient with a baseline renal glomerular filtration rate of 10.5 mL/min did not fulfill early safety criteria due to postinterventional acute kidney failure requiring temporary renal replacement therapy. At 30 days, there was one fatal event. Supplementary Table 2 (S3) provides patient‐level procedural and postprocedural data.

**TABLE 3:** In‐hospital outcomes and 30‐day mortality.

In‐hospital outcomes and 30‐day mortality		*n* = 30
Hospital stay, *days*	median (IQR)	8.5 (5.25 | 19.25)
Paravalvular leak	*n* (%)	
*none/trace*		28 (93.3)
*mild*		2 (6.7)
*moderate*		0 (0)
*severe*		0 (0)
New permanent pacemaker	*n* (%)	0 (0)
Stroke	*n* (%)	1 (3.3)
Device success	*n* (%)	28 (93.3)
Early safety	*n* (%)	27 (90.0)
30‐day mortality	*n* (%)	1 (3.3)

*Note:* The Summary of the in‐hospital outcomes and 30‐day mortality. Device success and early safety according to the VARC‐3 composite endpoint definitions.

Abbreviation: IQR—interquartile range upper/lower quartiles.

### 3.4 Survival

At a median follow‐up of 273 days (interquartile range 30–491 days, reverse Kaplan–Meier), overall survival was 93.3% (28/30). One patient died on postoperative day 2 and another on day 111 (cause of death unknown). Figure 2 displays a survival curve based on the Kaplan–Meier method. During that observation period, there were no device‐related rehospitalizations or reinterventions.

**FIGURE 2:**
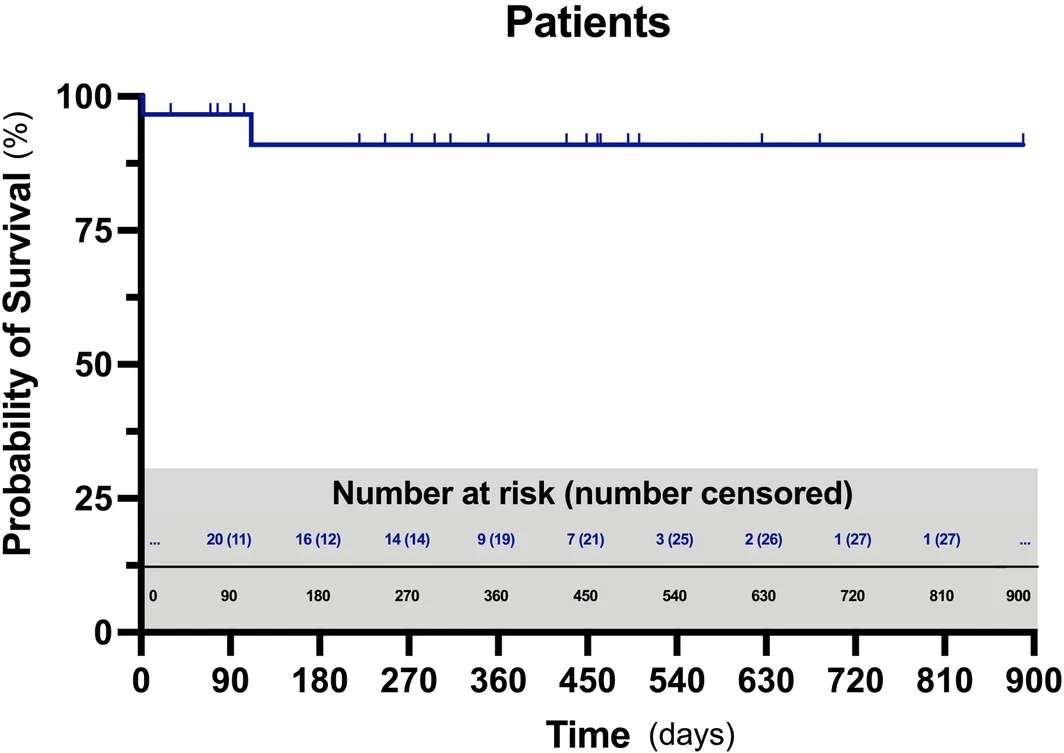
Kaplan–Meier survival curve of the cohort. Censored patients are marked as ticks on the survival curve. A number‐at‐risk‐table (gray area) is embedded within the curve figure with numbers at risk and numbers of censored subjects.

## 4 Discussion

This multicenter registry provides evidence that treatment of clinically relevant pure AR in LVAD patients with the JenaValve Trilogy is technically feasible and associated with favorable short‐term outcomes. Here, we give an update of a previous report from our registry [17]. Our analysis demonstrates that the device can be implanted safely in this challenging population, with higher rates of technical and device success compared to previous strategies, including non‐dedicated devices [11, 18, 19], and very low early mortality rates. These findings are particularly relevant given that conventional surgical treatment of AR in LVAD recipients carries prohibitive risk and off‐label use of standard TAVR systems has limited efficacy and safety.

The Trilogy system was specifically developed for non‐calcified anatomies, employing a locator‐based clipping mechanism that anchors the prosthesis to the native cusps independent of annular calcification. This feature directly addresses the anatomical challenges that have historically led to malposition, paravalvular regurgitation, or embolization with conventional transcatheter valves in the LVAD setting. Our study therefore extends prior case reports and single‐center experiences [17, 20, 21, 22, 23], showing consistent procedural success across geographically distinct, high‐volume institutions.

Although one‐third of the study population was supported with legacy LVAD platforms (HeartMate II or HVAD), these patients remain clinically relevant because they continue to require treatment for progressive aortic regurgitation during long‐term mechanical circulatory support. To facilitate interpretation of our findings in the context of current LVAD implantation practice, we additionally provide a complementary descriptive analysis of patients supported with the HeartMate 3 LVAD in Supplementary Appendix 3 (S4). The baseline, procedural, early clinical, and survival outcomes observed in the HeartMate 3 cohort appeared numerically similar to those of the overall cohort. As JenaValve Trilogy implantation is primarily determined by patient‐specific anatomy rather than the underlying LVAD platform, a direct mechanistic influence of LVAD type on procedural and related outcomes is unlikely. No formal statistical comparisons between LVAD platforms were feasible given the study design and sample size, therefore, the supplementary data should be interpreted as descriptive.

In the overall study cohort, survival was excellent, with only two deaths recorded during follow‐up. Although the low number of events limits the possibility for robust statistical inference, the absence of recurrent AR, valve embolization, or relevant paravalvular leak underscores the safety of this dedicated device in the LVAD population.

### 4.1 Limitations

This study has several limitations. First, its retrospective nature and lack of a comparator group limit interpretation. Second, the cohort size is small, which limits the statistical power of our analysis. Third, the VARC‐3 definition of device success incorporates assessment of intended valve performance. In our analysis, this criterion was primarily defined as effective elimination of aortic regurgitation. However, comprehensive evaluation of valve performance, including transvalvular gradients, can be challenging in LVAD patients due to limited echocardiographic windows and the frequent absence of native aortic valve opening. LVAD hemodynamics alter transvalvular flow, potentially limiting the applicability of standard VARC‐3 parameter cut‐offs. Furthermore, the study was conducted at experienced high‐volume centers, which may restrict the generalizability of results to institutions with less expertise in both LVAD and TAVR. As the participating U.S. centers contributed exclusively emergency‐use and compassionate‐use cases, which were not enrolled in the ongoing JENA‐VAD registry, a degree of selection bias toward more urgent and clinically complex patients cannot be excluded. While this is unlikely to affect procedural technical feasibility, it may confound short‐ and long‐term clinical outcomes. Hemodynamic follow‐up, including assessment of the effect of AR elimination on RV function, was not standardized across participating centers and was therefore not analyzed. However, no large‐scale prospective analyses have yet been conducted in this challenging and undertreated patient group Table 3.

## 5 Conclusions

In summary, our study demonstrates that the JenaValve system represents a safe and effective option for treating significant AR in LVAD patients at high surgical risk, with favorable short‐term outcomes.

## Author Contributions

H.H.: Concept/design, data collection, data analysis/interpretation, drafting the article, critical revision of the article, approval of the article. SY: Data collection, critical revision of the article, approval of the article. IL: Data collection, critical revision of the article, approval of the article. CK: Concept/design, data analysis/interpretation, critical revision of the article, approval of the article. EP: Concept/design, data analysis/interpretation, critical revision of the article, approval of the article. SG: Concept/design, data analysis/interpretation, critical revision of the article, approval of the article. DP: Concept/design, data analysis/interpretation, critical revision of the article, approval of the article. LR: Concept/design, data analysis/interpretation, critical revision of the article, approval of the article. AG: Concept/design, data analysis/interpretation, critical revision of the article, approval of the article. DK: Concept/design, data analysis/interpretation, critical revision of the article, approval of the article. RM: Concept/design, data analysis/interpretation, critical revision of the article, approval of the article. TV: Concept/design, data analysis/interpretation, critical revision of the article, approval of the article. AG: Concept/design, data analysis/interpretation, critical revision of the article, approval of the article. TR: Concept/design, data analysis/interpretation, drafting article, critical revision of the article, approval of the article. AU: Concept/design, data analysis/interpretation, drafting article, critical revision of the article, approval of the article.

## Conflicts of Interest

Isabell Just has received honoraria from AstraZeneca GmbH, Abbott GmbH, Abiomed Inc., and Biotest, unrelated to the submitted work. Christoph Klein is a proctor for JenaValve and has received speaker fees from Medtronic. Santiago Garcia is a consultant and proctor and has received institutional research grants from Edwards Lifesciences, Medtronic, Abbott Structural Heart, and Boston Scientific. Dean Kereiakes is a consultant to Edwards Lifesciences and J.C. Medical Inc. Lauren Ranard has received an institutional research grant from Boston Scientific and consulting fees from 4C Medical, InnovHeart, and Philips. Raj Makkar has received travel support from JenaValve. Torsten Vahl is reporting institutional funding to Columbia University Irving Medical Center from Boston Scientific, Edwards Lifesciences, JenaValve, and Medtronic, and he has received consulting fees from Abbott Vascular, Philips, and InnovHeart. Tanja Rudolph is a proctor and medical advisor for JenaValve. Axel Unbehaun is a proctor for Edwards Lifesciences, Medtronic, and JenaValve. All other authors have reported they have no relationships relevant to the contents of this paper to disclose.

## Supporting information


**Video 1** Supporting Information.

**Table 1** Patient‐level data summary on baseline demographic characteristics.

**Table 2** Patient‐level data summary of procedural and postprocedural outcomes.

**Table 3.1** HeartMate 3 subcohort characteristics.
Table 3.2 HeartMate 3 subcohort procedure characteristics.
Table 3.3 HeartMate 3 subcohort in‐hospital outcomes and 30‐day mortality.
Figure 3 HeartMate 3 subcohort survival. At a median follow‐up of 104 days (interquartile range 30–312 days, reverse Kaplan–Meier), overall survival was 100% (20/20). Figure 3 displays a survival curve for the HM3 subcohort based on the Kaplan–Meier method. During that observation period, there were no device‐related rehospitalizations or reinterventions.

## Data Availability

The data that support the findings of this study are available from the corresponding author upon reasonable request.
