# Supplementary material for: Early Multicenter Outcomes of Dedicated Transcatheter Aortic Valve Replacement for Left Ventricular Assist Device‐Associated Pure Aortic Regurgitation
Source: Artif Organs. 2026 Aug 25;50(10):1427–33. doi: 10.1111/aor.70226 (PMC13622895; doi:10.1111/aor.70226)
Supplement: Supplementary file 1 [file AOR-50-1427-s001.docx]

Supplementary Table 3.1. HeartMate 3 subcohort characteristics

| **HM3 subcohort characteristics** |  | **n = 20** |
| --- | --- | --- |
| Age, *years* | median (IQR) | 65 (58.0 \| 69.0) |
| Gender, *female* | n (%) | 6 (30.0) |
| BMI, *kg/m^2^* | median (IQR) | 26.5 (25.9 \| 29.2) |
| Time on LVAD, *years* | median (IQR) | 3.5 (1.0 \| 4.0) |
| EuroSCORE II, *%* | median (IQR) | 9.63 (8.34 \| 16.76) |
| NYHA functional class | n (%) |  |
| *I* |  | 0 (0.0) |
| *II* |  | 2 (10.0) |
| *III* |  | 8 (40.0) |
| *IV* |  | 10 (50.0) |
| Permanent pacemaker | n (%) | 14 (70.0) |

Table 1. Summary of the baseline cohort characteristics for all HeartMate 3 patients only. HM3 – HeartMate 3; IQR – interquartile range upper/lower quartile; BMI – body mass index; LVAD – left-ventricular assist device; NYHA – New York Heart Association.

Supplementary Table 3.2. HeartMate 3 subcohort procedure characteristics

| **HM3 subcohort procedure characteristics** | | **n = 20** |
| --- | --- | --- |
| Device size, *mm* | n (%) |  |
| 23/S |  | 2 (10.0) |
| 25/M |  | 7 (35.0) |
| 27/L |  | 11 (55.0) |
| Device access | n (%) |  |
| *transfemoral* |  | 19 (95.0) |
| *other* |  | 1 (5.0) |
| Anulus perimeter*, mm* | median (IQR) | 78.8 (74.6 \| 82.8) |
| Anulus average diameter, *mm* | median (IQR) | 24.9 (23.7 \| 26.3) |
| Anesthesia regimen | n (%) |  |
| *general anesthesia/intubation* |  | 14 (70.0) |
| *conscious sedation* |  | 6 (30.0) |
| Procedure duration*, min* | median (IQR) | 78.5 (59.0 \| 87.0) |
| Fluoroscopy duration*, min* | median (IQR) | 19.0 (16.0 \| 26.0) |
| Contrast volume, *ml* | median (IQR) | 93.5 (57.5 \| 115.0) |
| Technical success | n (%) | 19 (95.0) |

Table 2. Summary of the JenaValve Trilogy TAVI implantation procedural characteristics for all HeartMate 3 patients only. HM3 – HeartMate 3. IQR – interquartile range upper/lower quartile.

Supplementary Table 3.3. HeartMate 3 subcohort in-hospital outcomes and 30-day mortality

| **HM3 subcohort in-hospital outcomes and 30-day mortality** | | | **n = 20** |
| --- | --- | --- | --- |
| Hospital stay, *days* | median (IQR) | 9.5 (5.0\| 22.5) | |
| Paravalvular leak | n (%) |  | |
| *none/trace* |  | 20 (100) | |
| *mild* |  | 0 (0) | |
| *moderate* |  | 0 (0) | |
| *severe* |  | 0 (0) | |
| New permanent pacemaker | n (%) | 0 (0) | |
| Stroke | n (%) | 0 (0) | |
| Device success | n (%) | 19 (95.0) | |
| Early safety | n (%) | 18 (90.0) | |
| 30-day mortality | n (%) | 0 (0) | |

Table 3. Summary of the in-hospital outcomes and 30-day mortality for all HeartMate 3 patients only. Device success and early safety according to the VARC-3 composite endpoint definitions. HM3 – HeartMate 3. IQR – interquartile range upper/lower quartile.

Supplementary Figure 3. HeartMate 3 subcohort survival.

At a median follow-up of 104 days (interquartile range 30 – 312 days, reverse Kaplan–Meier), overall survival was 100 % (20/20). Figure 3 displays a survival curve for the HM3 subcohort based on the Kaplan-Meier method. During that observation period there were no device-related rehospitalizations or reinterventions.

Kaplan-Meier survival curve of the HM3 subcohort. Censored patients are marked as ticks on the survival curve. A number-at-risk-table (grey area) is embedded within the curve figure with numbers at risk and numbers of censored subjects.
