# Supplementary material for: Early Multicenter Outcomes of Dedicated Transcatheter Aortic Valve Replacement for Left Ventricular Assist Device‐Associated Pure Aortic Regurgitation
Source: Artif Organs. 2026 Aug 25;50(10):1427–33. doi: 10.1111/aor.70226 (PMC13622895; doi:10.1111/aor.70226)
Supplement: Supplementary file 2 [file AOR-50-1427-s002.docx]

| **Procedural parameters**  **and**  **Outcomes** | | Urgency | Anesthesia | Access | Annulus perimeter, *mm* | TAVR size, *mm* | Procedure duration, *min* | Procedural complication | Postprocedural complication | Paravalvular leak | New permanent pacemaker | Hospital stay, *days* | 30-day status |
| --- | --- | --- | --- | --- | --- | --- | --- | --- | --- | --- | --- | --- | --- |
| Patient | 1 | E | CS | TF | 82.5 | 27 | 148 | major vascular | none | <1 | No | 7 | alive |
|  | 2 | E | CS | TF | 74.0 | 25 | 79 | none | none | <1 | No | 5 | alive |
|  | 3 | E | CS | TF | 83.6 | 27 | 230 | minor vascular | none | <1 | No | 6 | alive |
|  | 4 | E | CS | TF | 78.9 | 25 | 67 | minor vascular | none | <1 | No | 15 | alive |
|  | 5 | E | CS | TF | 83.0 | 27 | 93 | none | none | <1 | No | 17 | alive |
|  | 6 | E | CS | TF | 74.7 | 25 | 60 | none | none | <1 | No | 9 | alive |
|  | 7 | U | ITN | TF | 77.0 | 27 | 50 | none | none | <1 | No | 1 | alive |
|  | 8 | E | ITN | TF | 73.6 | 25 | 50 | none | none | <1 | No | 6 | alive |
|  | 9 | U | ITN | TF | 67.0 | 23 | 60 | none | none | <1 | No | 3 | alive |
|  | 10 | U | ITN | TF | 82.9 | 27 | 50 | none | none | <1 | No | n/a | alive |
|  | 11 | U | ITN | TF | 85.1 | 27 | 119 | 2^nd^ THV as ViV | none | <1 | No | 22 | alive |
|  | 12 | E | ITN | TF | 74.4 | 25 | 60 | none | none | <1 | No | 13 | alive |
|  | 13 | E | ITN | TF | 75.7 | 23 | 56 | none | stroke | <1 | No | 2 | died |
|  | 14 | E | ITN | TF | 84.2 | 27 | 60 | none | none | 2 | No | 8 | alive |
|  | 15 | E | ITN | TF | 77.3 | 25 | 58 | none | none | <1 | No | 3 | alive |
|  | 16 | E | ITN | TF | 74.6 | 25 | 79 | none | none | <1 | No | 6 | alive |
|  | 17 | E | ITN | TF | 79.9 | 27 | 36 | none | none | <1 | No | 14 | alive |
|  | 18 | E | ITN | TF | 74.1 | 25 | 75 | none | none | <1 | No | 6 | alive |
|  | 19 | E | ITN | TF | 77.5 | 25 | 50 | minor vascular | none | <1 | No | 5 | alive |
|  | 20 | E | ITN | TF | 74.6 | 25 | 62 | none | none | <1 | No | 5 | alive |
|  | 21 | E | CS | TF | 78.6 | 27 | 90 | none | none | <1 | No | 3 | alive |
|  | 22 | U | CS | TF | 79.3 | 27 | 48 | none | none | <1 | No | 12 | alive |
|  | 23 | U | ITN | TF | 84.1 | 27 | 80 | none | AKI stage 4 | <1 | No | 20 | alive |
|  | 24 | E | ITN | TF | 84.4 | 27 | 36 | none | none | <1 | No | 7 | alive |
|  | 25 | E | ITN | TF | 91.2 | 27 | 44 | none | none | <1 | No | 5 | alive |
|  | 26 | U | ITN | TF | 81.0 | 27 | 81 | none | none | <1 | No | 40 | alive |
|  | 27 | U | ITN | TC | 71.5 | 23 | 78 | none | none | <1 | No | 42 | alive |
|  | 28 | U | ITN | TF | 83.0 | 27 | 63 | none | none | <1 | No | 46 | alive |
|  | 29 | U | ITN | TF | 74.0 | 25 | 126 | minor vascular | none | <1 | No | 111 | alive |
|  | 30 | U | ITN | TF | 79.0 | 27 | 84 | none | none | <1 | No | 23 | alive |

Supplementary Table 2. Patient-level data summary procedural and postprocedural outcomes. E – elective; U – urgent; CS - conscious sedation; ITN – intubation/general anesthesia; TF – transfemoral; TC – transcarotid; THV – transcatheter heart valve; ViV - valve-in-valve; AKI – acute kidney injury; n/a – not available;
