# Supplementary material for: Early Multicenter Outcomes of Dedicated Transcatheter Aortic Valve Replacement for Left Ventricular Assist Device‐Associated Pure Aortic Regurgitation
Source: Artif Organs. 2026 Aug 25;50(10):1427–33. doi: 10.1111/aor.70226 (PMC13622895; doi:10.1111/aor.70226)
Supplement: Supplementary file 3 [file AOR-50-1427-s003.docx]

| **Characteristics** | | Center | Age, *years* | Gender | BMI, *kg/m^2^* | Time on LVAD, *years* | Type of LVAD | EuroSCORE II, *%* | NYHA class | Comorbidities | GFR, *ml/min* | Permanent pacemaker |
| --- | --- | --- | --- | --- | --- | --- | --- | --- | --- | --- | --- | --- |
| Patient | 1 | GER 2 | 56 | F | 39.8 | 1 | HM 3 | 45.17 | III | HT, HLP, DM, CAD, Afib | 19.9 | Yes |
|  | 2 | GER 2 | 58 | F | 28.9 | 4 | HM 3 | 9.63 | III | HT | 25.4 | Yes |
|  | 3 | GER 2 | 66 | M | 20.3 | 7 | HW | 7.79 | III | HT, HLP, CAD, PAD, COPD | 24.6 | Yes |
|  | 4 | GER 2 | 48 | F | 26.9 | 2 | HM 3 | 3.71 | IV | HT, HLP, Afib | 91.6 | Yes |
|  | 5 | GER 2 | 63 | M | 35.5 | 4 | HM 3 | 11.96 | IV | HT, HLP, Afib | 42.0 | Yes |
|  | 6 | GER 2 | 65 | M | 29.0 | 7 | HW | 8.95 | II | CAD, PCI, Afib | 80.6 | Yes |
|  | 7 | US 3 | 74 | F | 28.0 | 4 | HM 3 | 9.60 | IV | HT, HLP, CAD, MI, PCI, PAD, Afib | 50.0 | Yes |
|  | 8 | US 2 | 83 | F | 27.7 | 7 | HM II | 18.00 | III | CAD, MI, PCI Afib | 50.0 | No |
|  | 9 | US 2 | 71 | M | 28.8 | 4 | HM 3 | 31.00 | IV | HT, CAD, MI, PCI, Afib | 41.0 | Yes |
|  | 10 | US 1 | 80 | M | 27.5 | 6 | HM II | 42.79 | IV | HT, HLP, CAD, MI, PCI, Afib | 36.0 | Yes |
|  | 11 | US 1 | 69 | M | 26.0 | 4 | HM 3 | 16.76 | III | HT, HLP, CAD, MI, PCI, Afib | 63.0 | Yes |
|  | 12 | GER 1 | 71 | F | 23.8 | 4 | HW | 13.10 | III | HT, Afib | 68.2 | No |
|  | 13 | GER 1 | 64 | F | 28.4 | 3 | HW | 10.70 | III | HT, CAD, MI, PCI, Afib, COPD | 59.7 | Yes |
|  | 14 | GER 1 | 73 | M | 23.7 | 11 | HM II | 8.90 | III | HT, HLP, MI, PCI, PAD, Afib | 36.5 | Yes |
|  | 15 | GER 1 | 75 | M | 29.4 | 4 | HM 3 | 8.70 | III | HT, HLP, CAD, MI, PCI | 31.7 | No |
|  | 16 | GER 1 | 58 | M | 26.2 | 1 | HM 3 | 8.34 | III | HT, HLP, CAD, MI, PCI, PAD, COPD | 55.0 | No |
|  | 17 | GER 1 | 66 | F | 27.7 | 3 | HW | 9.57 | III | HT, HLP, Afib | 77.0 | Yes |
|  | 18 | GER 1 | 41 | F | 20.8 | 4 | HW | 7.28 | III | HLP, Afib | 58.0 | No |
|  | 19 | GER 1 | 65 | M | 25.6 | 1 | HM 3 | 6.85 | III | HT, HLP, CAD, MI, PCI, Afib, COPD | 90.0 | Yes |
|  | 20 | GER 1 | 66 | M | 26.2 | 3 | HM 3 | 12.64 | III | HT, HLP, DM, CAD, MI, PCI | 62.6 | Yes |
|  | 21 | GER 2 | 69 | M | 26.5 | 5 | HM 3 | 5.72 | II | HT, HLP, CAD, MI, PCI, Afib | 31.0 | Yes |
|  | 22 | GER 2 | 66 | M | 25.8 | 1 | HM 3 | 12.61 | III | HT, HLP, Afib | 21.0 | Yes |
|  | 23 | GER 1 | 64 | M | 35.4 | 9 | HM3 | 17.47 | IV | HT, HLP, CAD, MI, PCI, Afib, COPD | 10.5 | Yes |
|  | 24 | GER 1 | 65 | M | 35.4 | 6 | HM3 | 17.01 | II | HT, DM, Afib | 27.3 | Yes |
|  | 25 | GER 1 | 84 | M | 24.4 | 9 | HW | 10.51 | III | Afib | 61.3 | Yes |
|  | 26 | US1 | 58 | M | 26.4 | 2 | HM3 | 28.99 | IV | CAD, MI, PCI, Afib, COPD | 61.1 | No |
|  | 27 | US1 | 32 | F | 26.4 | 0 | HM3 | 24.76 | IV | - | 50.0 | No |
|  | 28 | US1 | 48 | F | 25.0 | 3 | HM3 | 27.00 | IV | HT, HLP | 90.0 | No |
|  | 29 | US1 | 71 | M | 23.5 | 4 | HM3 | 29.94 | IV | HT, HLP, DM, CAD, MI, Afib, COPD | 54.0 | No |
|  | 30 | US1 | 67 | M | 17.1 | 0 | HM3 | 39.93 | IV | HT, DM, CAD, MI, PCI, COPD | 31.0 | Yes |

Supplementary Table 1. Patient-level data summary on baseline demographical characteristics. GER – Germany; US – United States. BMI – body mass index; LVAD – left ventricular assist device; NYHA – New York Heart Association; GFR – glomerular filtration rate; F – female; M – male; HM 3 – HeartMate 3; HM II – HeartMate II; HW – HeartWare; HT – hypertension; HLP – hyperlipoproteinemia; DM – diabetes mellitus; CAD – coronary artery disease; Afib – atrial fibrillation, PAD – peripheral artery disease; PCI – status post percutaneous coronary intervention; MI – status post myocardial infarction; COPD – chronic obstructive pulmonary disease.
